# Substitution of Poultry Fat with Black Soldier Fly (*Hermetia illucens*) Larvae Fat in Dog Diets: Effects on Digestibility, Palatability, Peroxidation of Dry Food, Immunity, Blood Biochemistry, and Faecal Characteristics of Adult Dogs

**DOI:** 10.3390/vetsci12040311

**Published:** 2025-03-29

**Authors:** Oğuzhan Kahraman, Fatma İnal, Mustafa Selçuk Alataş, Zekeriya Safa İnanç, Samed Damar, Ibrar Ahmed, Mustafa Uludağ, Tamer Çalıkoğlu

**Affiliations:** 1 Department of Animal Nutrition and Nutritional Diseases, Faculty of Veterinary Medicine, Selçuk University, 42250 Konya, Türkiye; fainal@selcuk.edu.tr (F.İ.); selcukalatas@selcuk.edu.tr (M.S.A.); zsafa.inanc@selcuk.edu.tr (Z.S.İ.); 2 Institute of Health Sciences, Selcuk University, Alaeddin Keykubad Campus, Yeni Istanbul Street No:335, 42130 Konya, Türkiye; samed.damar@selcuk.edu.tr; 3 Xiamen Creciente Agritech Co., Ltd., No. 482, Xiamen 361022, China; vetrao6@gmail.com; 4 Independent Researcher, Çamlıca District, 133. Street, 06200 Ankara, Türkiye; uludaghekim@gmail.com; 5 Independent Researcher, Çamlı District, Çamlıca Street, 35310 İzmir, Türkiye; tamercalikoglu@gmail.com

**Keywords:** black soldier fly larvae fat, digestibility, dog, immunity, lipid peroxidation, palatability, serum biochemistry, stool quality

## Abstract

This study evaluated the effects of partially or fully replacing poultry fat with black soldier fly larvae (BSFL) fat on faecal parameters, blood biochemistry, immune responses, nutrient digestibility, food preference, and lipid oxidation in dogs. A total of 18 adult Golden Retriever dogs (6 dogs per diet group) were subjected to a digestibility trial for 30 days. Three experimental diets were formulated: a control diet (6% poultry fat), BSF3 (3% poultry fat +3% BSF larvae fat), and BSF6 (6% BSF larvae fat). Serum biochemical parameters, including blood urea nitrogen, creatinine, AST, ALT, glucose, cholesterol, triglycerides, total protein, and immunoglobulin levels (IgE and IgG), were analysed from blood samples collected from all dogs. Faecal consistency scores were recorded over 5 days, and faecal pH, ammonia nitrogen (ammonia-N), short-chain fatty acids (SCFAs), and branched-chain fatty acid (BCFA) concentrations were determined during the final three days of the digestibility trial. Seven days after the digestibility trial, a modified two-bowl preference test was conducted over four days using 20 dogs to assess the palatability of the control and BSF6 diets. Lipid oxidation in the diets was evaluated by measuring thiobarbituric acid reactive substances (TBARSs, mg MDA/kg) at baseline (day 0) and after 2, 4, 7, and 10 months of storage at ambient temperature (23–25 °C). BSF6 exhibited the lowest dry matter and organic matter digestibility percentages (*p* < 0.05). Protein digestibility was reduced in both BSF3 and BSF6, while ether extract digestibility was significantly lower in BSF6 compared to the control diet (*p* < 0.05). The replacement of poultry fat with BSF larvae fat had no significant effect on serum biochemical parameters or IgE and IgG levels (*p* > 0.05). Faecal acetic acid concentrations were higher in the control group compared to BSF3 and BSF6, whereas faecal valeric acid concentrations were lower in the control group than in the BSF3 and BSF6 groups (*p* < 0.05). Although faecal pH and consistency scores were not significantly influenced by the dietary fat source, the total SCFA concentrations in faeces decreased with the substitution of BSF larvae fat (*p* < 0.05). Furthermore, the dogs demonstrated a higher preference rate for the control diet over the BSF6 diet (*p* < 0.05). In conclusion, the inclusion of BSF larvae fat in dry dog food formulations reduced nutrient digestibility and palatability but had no adverse effects on the health status of the dogs.

## Simple Summary

Black soldier fly (*Hermetia illucens*) is one of the most intensively studied insect species for use in animal nutrition. Despite the recognized nutritional potential of BSF larvae fat, there remains a lack of comprehensive scientific data regarding its effects on blood chemistry, immune function, nutrient digestibility, and the palatability of food in dogs. In this study, we aimed to evaluate the effects of replacing poultry fat with black soldier fly (BSF) larvae fat on faecal parameters, blood biochemistry, immune responses of dogs and their nutrient digestibility, food preference, and the lipid oxidation of foods. Three experimental diets were formulated: a control diet (containing 6% poultry fat), BSF3 (3% poultry fat +3% BSF larvae fat), and BSF6 (6% BSF larvae fat). BSF6 exhibited the lowest dry matter and organic matter digestibility. Although faecal pH and consistency scores were not significantly influenced by the dietary fat source, the total fatty acid concentrations in the faeces decreased with the substitution of BSF larvae fat with poultry fat. Also, BSF larvae fat reduced the protein and ether extraction digestibility and palatability of the diets. However, it had no adverse effects on the health status of the dogs.

## 1. Introduction

Fats and oils are supplemented in dog diets to increase dietary energy levels in order to meet daily nutrient requirements and palatability. Fats are considered an essential nutrient for dogs [1]. The rise in the number of pets globally has contributed to substantial growth in this sector, with the global pet food industry estimated to be worth approximately USD 115 billion. Dog and cat food account for approximately 70% of this value [2]. It has been reported that approximately 290,000 tonnes of animal fat is used each year to produce pet food in the USA, some of which is of human food grade [3]. With more than 155 million people in 55 countries facing a food crisis worldwide, competition for using ingredients in pet and human food significantly impacts global food security. Therefore, it is necessary to explore the potential of new sources of fats that are not currently used in human food [4].

The black soldier fly (*Hermetia illucens*) is one of the insect species most intensively studied for use in animal nutrition [5]. Major pet food brands worldwide have introduced formulas containing BSF protein, but there is still little interest in BSF lipids (fat). Recently, two European brands marketed eight pet foods containing BSF fat [4]. When producing partially or fully defatted larvae meal from black soldier fly larvae (BSFL), a significant amount (30–40%) of fat, a nutritionally valuable by-product, is obtained [6]. The fat content and fatty acid composition of BSFL are influenced by the feed used for their growth and the process of extracting the fat from larvae [7,8,9]. Approximately 90% of BSFL fat comprises six fatty acids: lauric; palmitic; oleic; linoleic; and, to a lesser extent, myristic and stearic acids. The most abundant is lauric acid, representing about a third of the total fatty acids [10].

BSFL fat not only fulfils the metabolic requirements of animals but also supports animal health. Lauric acid has a proven antimicrobial effect [11,12] and is also associated with many health benefits, such as the prevention of cardiovascular disease, antiviral properties, cancer prevention, and reducing the risk of Alzheimer’s disease and obesity [13]. Wang et al. [14] have shown that medium-chain fatty acids may be beneficial in reducing abdominal obesity compared to long-chain saturated or unsaturated fatty acids. Lauric acid offers specific benefits for the cognitive function of dogs: Chronic inflammation can negatively impact brain health in dogs, contributing to cognitive decline. Lauric acid exhibits anti-inflammatory properties, which may help protect neurons and support overall brain health. BSFL contains important levels of proteins and fats to nourish cats and dogs. BSFL also contains bioactive components, such as lauric acid, chitin, and antimicrobial peptides, known for their potential to improve animal health [15].

Insect fat, with a high lauric acid content, has been shown to positively affect digestive health in fast-growing broiler chickens [16,17]. Due to its lauric acid content, BSFL can regulate blood cholesterol levels [18]. In broiler chickens, BSFL fat significantly reduces serum total cholesterol and HDL compared to coconut oil [19]. Sypniewski et al. [20] reported that the serum total cholesterol, HDL, and LDL of young turkeys consuming BSF fat instead of soybean oil are significantly reduced. Furthermore, BSFL fat has been reported to positively influence plasma immune and antioxidant activity, as well as improve gut morphology and barrier integrity in broiler chickens [21].

The allergenic potential is the potential of a food or ingredient to cause sensitisation and allergic reactions, often in association with an IgE antibody. There is a risk of allergy to edible insects due to different rearing environments or acquisition methods. Many food components trigger IgE and IgG antibodies in patients without skin or gastrointestinal symptoms. Protein is one of the main allergens in dogs [22]. However, a decreased serum IgG concentration has been recorded with the addition of BSF fat in turkeys and broilers [23,24]. The pathogenic significance of IgG antibodies to food antigens in dogs has yet to be clarified; the conventional view is that their detection reflects only previous exposure and tolerance and not specific food-related pathogenesis.

The majority of research on insect fats has focused on consumption by poultry. Studies have shown no significant differences in nutrient digestibility when soybean oil was fully replaced with BSF fat in broiler diets [7,25]. Nevertheless, including BSF fat has been reported to enhance ileal crude protein digestibility and ileal digestible energy [23]. In most studies investigating substituting insect fat in broiler, laying hen, and turkey diets, no significant impact on crude protein and crude fat digestibility has been observed [24]. Similarly, diets containing BSFL fat do not affect nutrient digestibility, faecal quality, or blood parameters in dogs [26]. When BSFL fat was used to replace 8% of plant oil in the diets of healthy dogs, serum biochemical parameters remained within normal ranges, with no observed changes in body weight, nutrient digestibility, or short-chain fatty acid (SCFA) concentrations in faeces [27].

Over the past two decades, advances in human and companion animal immunology have facilitated a deeper understanding of the relationship between host nutrition, immunomodulation, and their impact on overall health. Given the adverse effects associated with high dietary levels of saturated fatty acids, such as palmitic and stearic acids, it is crucial to establish the optimal inclusion levels of insects as a dietary source of fatty acids. BSF is a highly investigated insect due to its strong fecundity, high conversion rate, high nutrition, low cost, and easy management, and it is most commonly used in pet food [28]. Despite the recognised nutritional potential of BSFL fat, there remains a lack of comprehensive scientific data regarding its effects on blood chemistry, immune function, nutrient digestibility, and the palatability of the food to dogs. Consequently, this study evaluates the impact of replacing half or all of the poultry fat in extruded dog food with BSFL fat on nutrient digestibility, faecal metabolites, faecal quality, selected biochemical and immunological blood parameters, food preference by dogs, and oxidative stability of the food.

## 2. Materials and Methods

### 2.1. Animals and Research Unit

The animal experiments were approved by the Selçuk University Faculty of Veterinary Medicine Experimental Animal Production and Research Center Ethics Committee, with the approval number 2022/69.

A total of 20, 12 male and 8 female, healthy Golden retriever dogs were subjected to trials (20 dogs underwent a preference test, and 18 dogs were used for a digestibility trial). They had a mean age of around 6 ± 2.1 years, with a body weight of 29 ± 3.43 kg and a mean body condition score of 6.0 ± 1.20 on a nine-point scale [29]. The dogs were treated for internal and external parasites. Fresh water was always available. The research was conducted in the Dog Research Unit of Prof. Dr. Hümeyra Özgen’s Research and Application Farm. This unit has 28 individual pens, with a concrete floor indoor area (190 × 190 cm) and an outdoor promenade area (510 × 230 cm).

### 2.2. Diets

**Figure 1:**
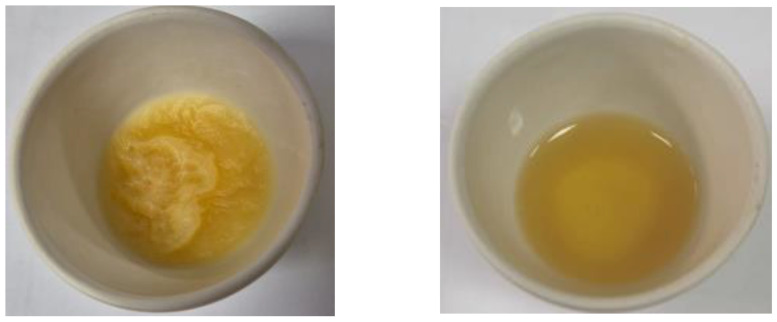
Black soldier fly larvae fat (solid form at ambient temperature (23–25 °C) on the left; liquid form melted by heating on the right).

The daily energy requirements of the dogs were determined from their activity levels and body weights. The metabolic energy contents of the produced dog foods were also calculated using Atwater factors. Three isonitrogenic diet formulations were prepared to meet the requirements of inactive adult dogs, according to the regulations of the European Pet Food Industry Federation [30]. The ingredients and determined chemical compositions of the diets are shown in Table 1. The experimental diets were manufactured at a feed production facility in Ankara, Türkiye. All raw materials used in the formulation of the diets were supplied directly by the factory. The ingredients were weighed according to the specified formulations and ground to pass through a 0.4 mm sieve. Following grinding, the ingredients were thoroughly homogenised in a mixer, after which water was added during the conditioning process to achieve a moisture content of 20–30%. The mixture was subsequently cooked for 4 min at temperatures gradually increasing from 90 °C to 135 °C. The conditioned mixture was processed through a DG-85 twin-screw extruder in four stages, producing wet extrudates that were then dried in a belt dryer at temperatures reaching up to 140 °C for 30–45 min. Poultry fat and solid larvae fat (Figure 1) were melted and sprayed onto the hot, dried kibble pellets. After cooling, the pellets were packed in airtight bags, with food samples collected from each bag for nutrient composition analysis before the bags were sealed.

### 2.3. Black Soldier Fly Larvae Fat

The BSFL fat used in this study was sourced from a private company in İzmir, Türkiye. Fatty acid analyses of the BSFL fat were performed at Zade Vital A.Ş. R&D Centre Laboratory (Konya, Türkiye), and the fatty acid composition of the BSFL fat is given in Table 2. The larvae were initially fed chick feed until reaching 7 days of age, after which their diet was transitioned to a mixture of dairy products, bread, and brewers’ barley. Once the larvae reached the prepupal stage (approximately 5%), they were harvested, washed, and euthanised by immersion in boiling water. The larvae were dried at 65 °C for 10 h and processed using a cold press oil extraction machine at 40 °C. The resulting solids were separated via centrifugation, and the extracted oil was siphoned off the surface and filtered through 30-micron filter paper to ensure purity.

### 2.4. Digestibility Trial

A total of 18 dogs were used in this experiment. The trial lasted 30 days; following 25 days of adaptation, faeces were collected from each dog for 5 days to determine individual nutrient digestibility and faecal characteristics. Dogs were randomly allocated into 3 groups (control, BSF3, and BSF6) of 6 dogs each, equalising body weight and gender. The food was fed to the dogs at a level of 3% above the maintenance energy requirements of inactive adult dogs [31]. The food was given at one meal and at the same time (9:30 am) every day.

The acid-insoluble ash (AIA) indicator method was used to determine digestibility [32]. For digestibility determination, 3–5 g of fresh faecal samples were collected from the concrete floor and placed in nylon bags daily for 5 days. Faecal samples were stored in a freezer (−18 °C) until analyses. Each dog’s faecal sample were thawed in ambient temperature and mixed. The pooled samples were then dried in an oven (VWR, Venti-line, Radnor, PA, USA) at 55 °C for 48 h. The dog food and faeces were ground using a laboratory mill (Retsch SM100, Haan, Germany) and passed through a 1 mm sieve. Analyses of acid-insoluble ash (AIA), dry matter (method 934.01), ash (method 942.05), crude protein (method 954.01), and ether extract (method 920.39) in foods and faeces were performed using AOAC methods [33]. Digestibility rates were calculated by the following equations:

Dry matter digestibility, % = 100 − 100 × (AIA in diet DM, %/AIA in faeces DM, %)

Nutrient digestibility, % = 100 − 100 × ((AIA in diet, % × Nutrient in faeces, %)/(AIA in faeces, % × Nutrient in diet, %))

### 2.5. Faecal Parameters

On the last 5 faecal collection days of the digestion trial, faeces were scored on a 1–5 scale by 3 experienced researchers [34], where 1 = pasty and shapeless stools; 2 = soft, malformed stools that take the shape of the collection container; 3 = soft, formed and moist stools that mark the floor; 4 = well-formed and consistent stools that do not mark the floor; and 5 = well-formed, hard and dry stools. Values between 3 and 4 were considered adequate and ideal. In the last 3 days, 3 g of fresh faecal samples collected daily for a maximum of 15 min after defecation were mixed with 30 mL of distilled water, and pH was measured with a digital pH meter (Hanna H183141) [35]. The short-chain fatty acid levels were determined in fresh faeces obtained up to 15 min after defaecation. Subsequently, 10 g of faeces was weighed in a labelled plastic jar with a cover and mixed with 30 mL of 16% formic acid. This suspension was blended and stored at 4 °C for three days. Before examination, the mixtures were centrifuged for 15 min at 5000× *g* (2 L21 centrifuge, Sigma, Osterodeam Hans, Germany) [36]. The levels of acetate, propionate, isobutyrate, butyrate, isovalerate, and valerate in the thawed samples were determined by gas chromatography (Agilent 6890N, Santa Clara, California, USA). For faecal ammonia, 2 g of fresh faeces was diluted with 1:5 distilled water, and 1 mL of the samples was mixed with 20 µL of sulphuric acid. The ammonia levels were then determined by a spectrometric method [37].

### 2.6. Serum Analysis

On the last day of the digestion trial, blood samples were taken from each animal, and sera were removed, and glucose, triglyceride, cholesterol, total protein, blood urea nitrogen (BUN), creatinine, aspartate aminotransferase (AST), and alanine aminotransferase (ALT) levels were measured by an autoanalyser at the Selçuk University Animal Hospital Laboratory of the Faculty of Veterinary Medicine. Immunoglobulin levels in serum samples were determined using canine IgG and IgE ELISA kits [38].

### 2.7. Preference Test

Two extruded diets, one containing 6% poultry fat (control) and the other containing 6% BSFL fat (BSF6), were evaluated for palatability using a modified two-bowl preference test for four days with 20 adult Golden Retriever dogs. The preference test began seven days after the digestibility test. During these 7 days, the dogs were fed different commercial dry foods to ensure they forgot the taste and odour of the experimental diets. This methodology was adapted from the two-bowl preference test described by Cabrita et al. [39], which assesses the food first approached and tasted. This modified approach was designed to optimise time and minimise food waste. Unlike traditional preference tests, this method does not require collecting or weighing leftover food. During the test, conducted after a 16 h fasting period, only the number of movements made by the dogs toward the food bowls were recorded. A total of five movements per test session were counted. The two diets (control and BSF6) were presented to the dogs in identical bowls containing 200 g of food, corresponding to approximately 65% of their daily energy requirements. If a dog consumed the first food offered in its entirety without switching to the second bowl, it was considered to have a 100% preference for that food. To account for potential side biases, the position of the bowls was alternated daily. After the five movements were recorded, the dogs were allowed to consume both diets freely. The preference rate was calculated using the following equation:

Preference ratio = (number of moves × 100)/5

### 2.8. Determination of Peroxidation of Dry Foods

Samples of the 3 foods produced for this research were stored at ambient temperature (23–25 °C) and were protected from air and light for 10 months. The thiobarbutyric acid (TBARS, mg MDA/kg) value of the foods was determined on the 1st (day 0), 2nd, 4th, 7th, and 10th months after production [40].

### 2.9. Statistical Analysis

A normality test was applied to the data obtained from the digestibility test, preference test, and oxidation measurements in SPSS v.23 (IBM Corp., Armonk, NY, USA). In the normality test, data with a significance level greater than 0.05 in the Shapiro–Wilk test, with skewness and kurtosis values distributed between −2 and +2, or with skewness and kurtosis values divided by the standard error, with a distribution between −1.96 and +1.96, were checked. The data that conformed to two of the above three evaluations were accepted as normal.

GLM was applied to the normally distributed data, and the Bonferroni test was applied to evaluate the differences. In addition, linear and quadratic effects were obtained using polynomial contrasts. Oxidation measurements were evaluated by GLM univariate analysis by selecting measurement time as the fixed factor, repeated measurements as the contrasts, and Bonferroni for differences. The significance of the fat preferences of the animals was determined by a paired samples *t*-test.

## 3. Results

The dry matter, organic matter, crude protein, and fat digestibility levels determined by the indicator method are given in Table 3. Dry matter and organic matter digestibility percentages were lowest in BSF6 (*p* < 0.001). The crude protein digestibility of BSF6 was lower than the control (*p* < 0.001). The highest digestibility percentage of the ether extraction was determined in the control food (*p* = 0.002).

Biochemical parameters measured in blood samples collected from all animals on the final day (30th day) of the digestibility trial, before the morning feeding, are presented in Table 4, and blood IgE and IgG levels are shown in Table 5. No significant differences were observed in serum glucose, triglyceride, cholesterol, blood urea nitrogen (BUN), total protein, creatinine, aspartate aminotransferase (AST), or alanine aminotransferase (ALT) levels between the groups. Similarly, including poultry fat or BSFL fat in the diets had no significant effect on serum IgE or IgG concentrations (*p* > 0.05).

The faecal scores of the faeces of all animals on the last five days of the digestion trial were visually scored by three researchers. The pH, dry matter, ammonia nitrogen, and SCFA of fresh faecal samples collected on the last three days of the digestion trial are given in Table 6. The faecal acetic acid level was higher in the control-group dogs (*p* = 0.005). Although the amount of isovaleric acid was lower in the BSF3-group dogs, the lowest valeric acid levels were measured in control-group dog faeces (*p* = 0.037).

The preference test results are presented in Table 7. The BSF6 food was preferred at the rate of 55.13%. This percentage was significantly higher than the preference rate of the BSF6 food (44.88%) (*p* = 0.035).

**Figure 2:**
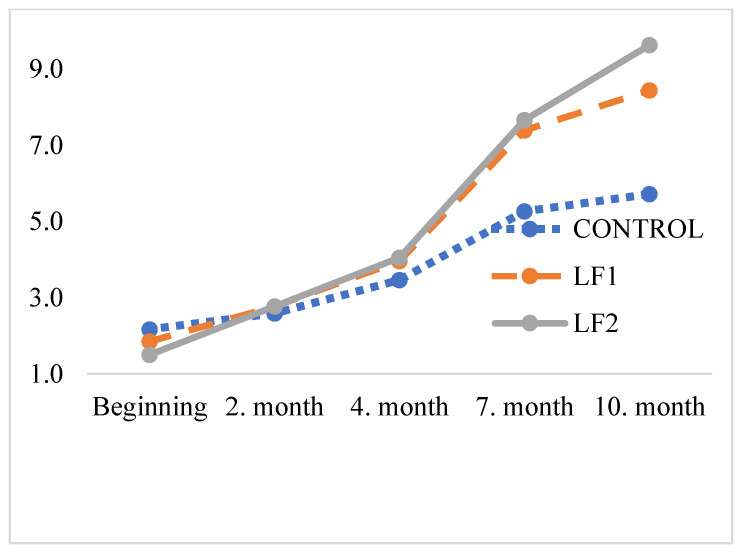
TBARS values of the foods stored for different periods, mg MDA/kg.

The results of MDA levels of 0, 2, 4, 7, and 10 months after storage of the foods are presented in Table 8, and corresponding curves are shown in Figure 2. Similar oxidation values were observed in dog foods containing poultry fat and BSFL fat throughout the 10-month storage period.

## 4. Discussion

Although an equal amount of fat was added to all three foods, chemical analyses showed that the ether extract was lower in the foods where BSFL was used. In the chemical analysis of BSFL, 99.79% dry matter and 94.22% ether extract were measured. After the fat was obtained, the protein was separated along with the sticky substances in the purification process. A previous study measured 92% ether extract in BSFL [41].

Martins et al. [42] reported that replacing linseed oil with BSFL fat at 3% and 6% in the diets of young rabbits negatively affected the digestibility of dry matter, organic matter, and crude fat, as observed in this study. However, another study showed that replacing soybean oil with BSFL fat did not impact nutrient digestibility [43]. This discrepancy is likely due to the lower levels of BSFL fat (1.5%) in the study by Gasco et al. [43]. In contrast to these findings, Freel et al. [30] reported no differences in the digestibility of dry matter and crude protein between diets containing poultry fat and those containing BSFL fat for dogs. Freel et al. [27] also observed higher digestibility coefficients for dry matter (90–91%), crude protein (89–91%), and ether extraction (96.0–96.8%) of dry foods compared to the results of this study (74.5–81.2%, 73.9–79.9%, and 86.3–93.9%, respectively). Similarly, Jian et al. [28] found that the inclusion of BSFL fat in dog diets reduced the dry matter digestibility (from 82.2% to 78.9%) and ether extract digestibility (from 95.5% to 93.9%), although these reductions were not statistically significant. Kierończyk et al. [7] reported that replacing soybean oil with BSFL fat did not affect ether extract and crude protein digestibility in broiler chickens.

In contrast, another study by Kierończyk et al. [23] demonstrated increased crude protein digestibility in broilers fed diets containing BSFL fat. These variations in digestibility results can likely be attributed to differences in the nutritional composition and fatty acid profiles of the BSFL fat, which are influenced by the environmental conditions in which the larvae were reared. The reduced nutrient digestibility in diets containing larvae fat in this study may be due to the inhibitory effect of lauric acid on gut microbiota [44]. Insect fat, which is normally rich in medium-chain fatty acids, is expected to be absorbed more than poultry fat, which contains more long-chain fatty acids. However, the triacylglycerol composition of BSFL fat and the distribution of lauric acid in triacylglycerols may have adversely affected digestibility. In addition, it has been reported that the high melting point of saturated fatty acids, as in BSFL fat, makes them less soluble in bile, reducing the viscosity of the intestinal contents and their digestibility in the intestine [45].

The biochemical results obtained in this study are consistent with the studies in dogs, rabbits, fish, and broilers [23,25,28,43,46]. Because there is no standard for the diet of BSFLs and their fatty acid composition may vary considerably, BSFL fat used in different studies may affect cholesterol levels or different parameters differently in animals.

Acetate is the major SCFA in the colon and has been shown to increase cholesterol synthesis after absorption. However, propionate has been shown to inhibit cholesterol synthesis. Therefore, substrates that can reduce the acetate:propionate ratio may reduce the risk of cardiovascular disease. As long as the faecal quality is not affected, it is preferable to produce more SCFAs [39]. Aside from its effects on health, the low intestinal acetic acid level in dogs consuming BSF fat-based food is an unfavourable condition. Acetic acid regulates the pH balance in the intestine and aids digestion. Low levels can negatively affect digestion and impair nutrient absorption [47]. This could be another reason for the reduced nutrient digestibility of the BSF3 and BSF6 dry foods.

The decreased acetate level may be due to lauric acid in BSFL fat suppressing acetic acid-producing bacteria by changing the intestinal microbiota [48]. *Clostridiaceae*, which break down indigestible complex carbohydrates to produce SCFAs, may have been negatively affected by the presence of lauric acid [49]. Consistent with this study, Kierończyk et al. [50] and Jian et al. [28] also reported propionate and butyrate levels unaffected by BSFL fat administration. Valeric acid was reported to increase in the faecal content of broiler chickens fed larvae fat [51]. A previous study highlighted numerous protective and beneficial effects associated with the increase in or administration of valeric acid, including its potential role in mitigating allergies, intestinal dysbiosis, colitis, enteritis, experimental encephalitis, and eczema [49].

Ammonia in faeces is a putrefactive by-product generated through the fermentation of undigested protein. In the present study, faecal ammonia concentrations increased for dogs fed diets containing BSFL fat as a replacement for whole-poultry oil, which aligns with the reduced protein digestibility of these diets. Faecal pH is strongly correlated with SCFAs and is a reliable indicator of SCFA production. Within the intestine, microbial fermentation of substrates produces SCFAs, lowering luminal pH and creating an acidic environment that inhibits the proliferation of pathogenic bacteria. A reduction in butyrate levels typically increases pH. Faecal pH is also regarded as a marker of faecal health [52].

This study hypothesised that the faecal pH would be lower in the groups fed BSFL fat due to the observed lower total SCFA concentrations. However, no decrease in faecal pH was detected in these groups, likely due to elevated faecal ammonia levels. Despite these differences, faecal pH remained within normal physiological limits. Similarly, Jian et al. [28] reported no significant changes in faecal pH in dogs fed diets containing BSFL fat as a replacement for poultry fat, which agrees with the findings of the present study. In this study, the crude BSFL fat used in the diets was noted to have an unpleasant odour compared to poultry fat. Previous research has indicated that various residues and components in larval fat can influence its flavour [11]. Understandably, dogs prefer poultry fat, which they are accustomed to, over the unfamiliar larval fat. Additionally, the higher linoleic acid content in poultry fat compared to larval fat may also influence the preference of dogs [1]. Although Freel et al. [27] have reported that dogs readily accepted BSFL fat, their study measured the consumption of larval fat-containing diets without directly comparing them to a control group. Furthermore, the larval fat used in their study contained 38.4% lauric acid and 17.5% linoleic acid, whereas the BSFL fat in the present study contained 42.84% lauric acid and 9.90% linoleic acid. In a study by Schiavone et al. [17], no significant differences were observed in the preference of broiler chickens between soybean oil and BSFL fat, further highlighting the variability in fat preferences across species.

Currently, there is no established limit for MDA levels in dog foods. In this study, the MDA levels of the poultry oil and BSFL fat were not analysed prior to their incorporation into the diets. However, measurements conducted immediately after food production gave an initial MDA value of 1.84 mg/kg, suggesting that the MDA levels in the oils could be substantially higher. Given the variability in measurement methods and the wide range of reported values in the literature, determining whether the diets were oxidised remains challenging. For instance, Larouche et al. [52] reported an MDA concentration of 2.8 mg/kg in BSFL dried at 60 °C for 30 min prior to degreasing. Similarly, Zhen et al. [53] observed MDA levels exceeding 3.6 mg/kg in dried larvae using various methods prior to degreasing and storage for 1 month. In the current work, malondialdehyde levels increased from 2.17 to 5.72 mg/kg (2.6 times) in the control group and from 1.50 to 9.63 mg/kg (6.4 times) in the BSF6 group after 10 months.

## 5. Conclusions

Including BSFL fat in extruded dog food and replacing half (3%) or all (6%) of the poultry oil during production did not have adverse health effects. Reduced nutrient digestibility was observed, and faecal scores remained unaffected. However, the faecal SCFA levels of the dogs that consumed BSFL fat-containing food reduced. This issue could potentially be mitigated by purifying larval fat and gradually acclimatising dogs to this novel dietary ingredient. The findings of this study suggest that BSFL fat cannot serve as a viable alternative fat source in dog food formulations. Also, high input amounts and costs currently hinder the widespread adoption of insect products. If vegetable oils and animal fats used in dog food formulations become more expensive, insect fats could be considered an alternative. However, the fatty acid profile that differentiates BSFL from other animal or plant products must be considered when formulating dog diets. Future studies should focus on determining the methods of extraction and the nutritional profile of BSFL fat intended for inclusion in pet food. Efforts should be made to minimise exposure to heat treatment, incorporate the fat in its freshest form, and measure primary oxidation products prior to inclusion. Additionally, oxidation parameters should be monitored in animals consuming these diets. Further research is needed to evaluate the effects of including BSFL fat in dry food on digestibility, intestinal fermentation products, health, and immune function in greater detail. Strategies to enhance the palatability of BSFL fat-containing extruded foods should also be explored.

## Figures and Tables

**Table 1:** Ingredient list and chemical composition of control and experimental diets.

Ingredients	Control%	BSF3%	BSF6%
Corn	35.0	35.0	35.0
Barley	20.0	20.0	20.0
Rice	15.0	15.0	15.0
Poultry meal	15.0	15.0	15.0
Corn gluten meal	6.4	6.4	6.4
Poultry fat	6.0	3.0	
Black soldier fly larvae fat		3.0	6.0
Dehydrated whey	2.0	2.0	2.0
Vitamin–mineral mixture *	0.35	0.35	0.35
Potassium chloride	0.25	0.25	0.25
Calculated chemical composition, 100 g DM			
Energy, kcal	430.9	431.2	432.2
Protein, g	21.5	21.6	21.7
Fat, g	9.5	9.7	9.8
Fiber, g	2.9	3.2	3.6
Carbohydrates, g	64.5	65.2	64.8
Calcium, g	0.6	0.6	0.6
Phosphorus, g	0.6	0.6	0.6
Determined chemical composition			
Metabolisable energy, kcal/kg	3759.1	3647.2	3632.1
Dry matter, %	96.4	94.9	94.8
Crude protein, %DM	20.27	20.6	20.9
Acid hydrolised ether extraction, %DM	11.3	10.3	10.7
Crude fiber, %DM	5.5	5.4	5.8
Ash, %	6.3	5.8	5.9

BSF3: a diet formulated by replacing 50% of the poultry fat in the control diet with black soldier fly larvae fat; BSF6: a diet in which black soldier fly larvae fat fully replaced the poultry fat present in the control diet. * mg/kg: biotin: 20; choline: 200,000; cyanocobalamin: 6; folate: 400; niacin: 16,000; pantothenic acid: 2800; pyridoxine: 2000; riboflavin: 2000; thiamine: 1200; vit A: 432; cholecalciferol: 2.5; vit E: 8000; Cl: 100,000; Cu: 2000; Fe: 24,000; I: 400; K: 300,000; Mg: 50,000; Mn: 40,000; Se: 40; Zn: 24,000.

**Table 2:** Fatty acid composition of BSF larvae fat.

Fatty Acids		%
10:0	Capric acid	0.69
12:0	Lauric acid	42.84
14:0	Myristic acid	8.04
14:1	Myristoleic acid	0.23
16:0	Palmitic acid	17.02
16:1	Palmitoleic acid	3.19
17:1	cis-10-Heptadecanoic acid	1.94
18:1	n9 cis Oleic acid	14.60
18:2	n6 cis Linoleic acid	9.90
18:3	n3 alpha Linolenic acid	0.12
20:0	Arachidic acid	0.65
20:1	n9 Gondoic acid	0.26
20:2	cis-10.14-Eicosadienoic acid	0.32
22:1	n9 Erusic acid	0.15
24:0	Lignoceric acid	0.05

**Table 3:** Nutrient digestibility percentages of control and experimental dry foods.

Item	Control	BSF3	BSF6	SEM	*p*-Value
Dry matter digestibility, %	81.17 ^a^	80.03 ^a^	74.51 ^b^	0.81	<0.001
Organic matter digestibility, %	84.91 ^a^	83.58 ^a^	79.36 ^b^	0.75	<0.001
Crude protein digestibility, %	79.88 ^a^	77.62 ^ab^	73.93 ^b^	1.23	0.013
Ether extract (fat) digestibility, %	93.91 ^a^	86.30 ^b^	87.58 ^b^	1.30	0.002

*n* = 6; ^a,b^ means in the same row with different superscripts differ significantly. Control: diet formulated with 6% poultry fat; BSF3: a diet formulated by replacing 50% of the poultry fat in the control diet with black soldier fly larvae fat; BSF6: a diet in which black soldier fly larvae fat fully replaced the poultry fat present in the control diet.

**Table 4:** Comparison of blood biochemical values of dogs fed poultry fat and BSF larvae fat containing foods.

Item	Reference Range	Control	BSF3	BSF6	SEM	*p*-Value
BUN, mg/dL	6–31	15.80	19.27	16.67	1.46	0.248
Creatinine, mg/dL	0.5–1.6	1.10	1.20	1.08	0.09	0.636
AST, U/L	15–66	23.00	26.83	20.67	1.80	0.081
ALT *, IU/L	12–118	20.17	24.83	23.17	2.45	0.416
Glucose, mg/dL	70–138	114.50	109.67	111.83	2.77	0.483
Cholesterol, mg/dL	92–324	308.17	266.00	247.50	24.08	0.222
Triglyceride, mg/dL	29–291	38.17	32.00	36.83	3.83	0.504
Total protein, mg/dL	5.0–7.4	5.80	5.67	5.98	0.18	0.479

*n* = 6; * Kruskal–Wallis test; Chi Square: 3399; df: 2; SEM: standard error of the mean; control: diet formulated with 6% poultry fat; BSF3: a diet formulated by replacing 50% of the poultry fat in the control diet with black soldier fly larvae fat; BSF6: a diet in which black soldier fly larvae fat fully replaced the poultry fat present in the control diet.

**Table 5:** Serum immunoglobulin levels of dogs fed poultry fat and BSF larvae fat containing foods.

Item	Group	Mean	SEM	*p*-Value
Immunoglobulin E, µg/mL	Control	1.11	0.19	0.129
	BSF3	0.55		
	BSF6	1.04		
Immunoglobulin G, µg/mL	Control	386.41	68.54	0.483
	BSF3	433.18		
	BSF6	313.52		

SEM: standard error of the mean; control: diet formulated with 6% poultry fat; BSF3: a diet formulated by replacing 50% of the poultry fat in the control diet with black soldier fly larvae fat; BSF6: a diet in which black soldier fly larvae fat fully replaced the poultry fat present in the control diet.

**Table 6:** Effects of BSF larvae fat on some faecal parameters.

Parameter	Control	BSF3	BSF6	SEM	*p*-Value
Acetic acid, µmol/g DM	348.44 ^a^	292.68 ^b^	287.92 ^b^	11.99	0.005
Propionic acid, µmol/g DM	139.53	136.91	132.52	10.37	0.891
Isobutyric acid, µmol/g DM	6.26	5.20	6.17	0.38	0.132
Butyric acid, µmol/g DM	54.12	44.19	50.56	6.75	0.585
Isovaleric acid, µmol/g DM	6.92 ^a^	5.13 ^b^	7.03 ^a^	0.52	0.034
Valeric acid, µmol/g DM	2.63 ^b^	5.22 ^a^	5.47 ^a^	0.77	0.037
TSCFA, µmol/g DM	557.90 ^a^	489.32 ^b^	489.68 ^b^	18.31	0.027
NH_3_-N, µmol/g DM	102.51 ^b^	106.67 ^ab^	111.48 ^a^	1.61	0.005
Faecal pH	6.82	6.84	7.07	0.13	0.329
Faecal score	3.86	3.76	3.79	0.04	0.268

*n* = 6; ^a,b^ means in the same row with different superscripts differ significantly (*p* < 0.05); TSCFAs: total short-chain fatty acids; SEM: standard error of the mean; control: diet formulated with 6% poultry fat; BSF3: a diet formulated by replacing 50% of the poultry fat in the control diet with black soldier fly larvae fat; BSF6: a diet in which black soldier fly larvae fat fully replaced the poultry fat present in the control diet.

**Table 7:** The results of dogs’ preference rates between diets containing poultry fat and those containing black soldier fly larval fat.

Group	*n*	Preference Rate, %	SEM	*p*-Value
Control	20	55.13	2.25	0.035
BSF6	20	44.88		

SEM: standard error of the mean; BSF6: a diet in which black soldier fly larvae fat fully replaced the poultry fat present in the control diet.

**Table 8:** Values of thiobarbituric acid reactive substances (TBARSs) determined at different storage times in foods.

Time	*n*	TBARSs, mg MDA/kg			
		Control	BSF3	BSF6	Overall
Day 0	3	2.17 ± 0.04	1.85 ± 0.02	1.50 ± 0.06	1.84 ± 0.10
2 month	3	2.59 ± 0.09	2.77 ± 0.05	2.77 ± 0.05	2.71 ± 0.04
4 month	3	3.46 ± 0.09	3.96 ± 0.03	4.05 ± 0.04	3.82 ± 0.10
7 month	3	5.27 ± 0.25	7.39 ± 0.03	7.66 ± 0.09	6.77 ± 0.39
10 month	3	5.72 ± 0.09	8.44 ± 0.24	9.63 ± 0.14	7.93 ± 0.58
Overall	15	3.84 ± 0.38	4.88 ± 0.69	5.12 ± 0.82	
Tests of between-subject effects					
Sources	Sum of squares		df	Mean of squares	*p*-value
Time	248.06		4	62.01	<0.001
Group	13.92		2	6.96	0.139
Time × Group	21.86		8	2.73	<0.001

TBARSs: thiobarbituric acid reactive substances; MDA: malondialdehyde; LF1: BSF larvae fat, 3%; LF2: BSF larvae fat, 6%; df: degrees of freedom.

## Data Availability

The data presented in this study are available upon request from the corresponding author.
